# Self-disclosure by adolescents in therapy for eating difficulties: A Q-Methodology study

**DOI:** 10.1177/13591045231209648

**Published:** 2023-10-25

**Authors:** Rhiannon Dunlop, Laura M Simonds, Mary John

**Affiliations:** 1 Department of Psychological Interventions, 3660 University of Surrey, UK; 2 Solent East CAMHS Eating Disorder Service, 232267 Solent NHS Trust, UK

**Keywords:** Self-disclosure, eating disorders, eating difficulties, adolescents, q-methodology, therapy

## Abstract

Facilitating client self-disclosure is essential to therapeutic effectiveness. Given the long-term consequences of eating difficulties for adolescents, there is a need for more research on self-disclosure in this group. This study investigated factors likely to influence adolescents’ decisions to self-disclose during psychological therapy for eating difficulties using Q-methodology. Participants (*n* = 28), recruited through child and adolescent mental health services in the UK, completed a task that involved sorting 47 statements to represent their viewpoint on self-disclosure. The 28 completed sorts were subjected to a by-participant factor analysis in order to identify distinct viewpoints in the sample. Three distinct factors were extracted. One factor emphasised the importance of therapist self-disclosure on decisions to disclose. In contrast, another factor placed more emphasis on the influence of eating disorder identity and readiness to change on disclosure decisions. The third factor placed emphasis on the quality of the therapeutic relationship and readiness to change as having most influence. Given the absence of a unifying factor representing what influences the decision to disclose, clinicians should ensure they explore with young people what might influence their decision to disclose.

Eating disorders and eating difficulties (ED) that do not meet the threshold for a formal diagnosis are prevalent within adolescent populations (Swanson et al., 2011). A substantial corpus of literature indicates that non-disclosure is an inherent aspect of eating disorders. Phenomenological research provides insight into why non-disclosure might be so ubiquitous. For example, young people with anorexia can believe that it is their only way of coping with unpleasant emotions and experiences; focussing thought on food and eating can allow avoidance and numbing of negative emotions (Williams & Reid, 2012). A strong sense of identity may be built around control of food, weight, and shape, particularly during the uncertain period of adolescence as young people attempt to individuate within a social context permeated with ideals around appearance; relinquishing this control amounts to an existential threat (Bryant et al., 2022). Understanding factors that influence disclosure is vitally important in facilitating the early recognition of disordered eating, as well as the efficacy and effectiveness of early interventions. The objective of this study was to explore factors that adolescents with EDs perceive to influence their decision to self-disclose in therapy.

Several factors might impact self-disclosure in therapy for EDs. Denial and reduced perception of the severity of difficulties have frequently been identified as barriers to seeking help, which is likely to influence disclosure (Ali et al., 2017; Becker et al., 2004; Cachelin et al., 2001; Gratwick-Sarll et al., 2016). Examination of barriers to young adults with EDs seeking help indicates that embarrassment, fear of treatment, change, and a belief that treatment would not work are some of the factors (Ali et al., 2020). The literature to date has placed limited focus on self-disclosure or help-seeking barriers in the adolescent population, despite adolescence being a pivotal point for targeting prevention and treatment for such difficulties.

Whilst the literature does not specifically focus on disclosure in adolescents, research exploring factors that might make disclosure for adults with an ED difficult has focused on its relationship with shame (Becker et al., 2010; Simonds & Spokes, 2017; Swan & Andrews, 2003). Dayal et al. (2015) suggested that not disclosing may protect the individual from further shame. The reactions of others (Becker et al., 2010), as well as shame associated with eating behaviours (Swan & Andrews, 2003), have also been shown to be barriers to disclosure. This is consistent with research suggesting that it is the stigmatising attitudes of others that prevents help-seeking (Akey et al., 2013; Becker et al., 2010). It is likely that stigma and shame negatively impacts disclosure (Simonds & Spokes, 2017). Shame associated with disclosing aspects of EDs may leave individuals feeling vulnerable and deprived from the safety and protection that keeping their ED hidden may provide. Furthermore, disclosure may lead to an emergence of painful memories, thoughts, or emotions that may make it harder to stay in control (Farber, 2006). This is supported by Ali et al. (2017) who found fear of losing control, and of change, to be significant barriers to help seeking for EDs.

Several authors have drawn attention to the potential significance of identity in EDs, for example, that anorexia arises from a “lost sense of self” (Oldershaw et al., 2019) or that it provides a sense of identity (Nordbø et al., 2006). Oldershaw et al.’s (2019) conceptualisation of anorexia suggests that it emerges as a way of managing overwhelming emotions and creates a ‘false sense of self’. This may make self-disclosure for young people with EDs more challenging as it could be perceived as ‘letting go’ of the identity that gives them meaning. In a Q-sort study with an adult sample, Murphy et al. (2020) found that wanting to maintain an ED identity influenced some participants’ decisions to disclose during therapy, although this was not influential for all participants.

Aspects of the therapeutic alliance have also been considered to have a possible impact on disclosure. Swan and Andrews (2003) found that 39% of participants had not disclosed information about their EDs during therapy because of an issue within the therapeutic relationship including not trusting the therapist, fear of being judged, and not wanting to let go of some control within the therapeutic relationship. There is limited and rather mixed research on the impact of therapist self-disclosure. Whilst some studies have indicated that therapist self-disclosure of personal experience of ED can enhance the therapeutic alliance (de Vos et al., 2016; Patmore, 2020), and that the extent to which clients perceive therapist self-disclosures to be helpful predicts enhanced alliance and subsequent client self-disclosure (Simonds & Spokes, 2017), other studies suggest personal therapist self-disclosure is perceived to be inappropriate (Oyer et al., 2016; Skytte-Glue & O’Neill, 2010).

Taken together, the foregoing discussion indicates several factors that might influence decisions to disclose during therapy for an ED. Research to date focuses on the perceptions of adults; these cannot be assumed to apply to adolescents due to their different stage of life and development. The current study addressed this gap by exploring what adolescents perceive influences their decision to self-disclose during psychological therapy for EDs.

## Method

### Design

The study was granted ethical approved by South Central Hampshire A NHS Research Ethics Committee. Choice of design was influenced by the lack of research in this participant group, as well as the potential difficulties of gaining disclosures about a topic that is often denied. Taking an approach that allowed for participant subjectivity was considered important given the lack of research with adolescents. Of particular importance was utilising a methodology that allowed for the nuances of self-disclosure decisions to be revealed to increase the study’s theoretical and practice implications. Interviews were not considered to be optimal given the pressures on self-presentation during in-person data collection. This method might also have limited the sample size. Taken together, these considerations suggested a Q-methodology would be most effective.

Q-methodology allows for subjective viewpoints on a topic to be identified by asking participants to sort predetermined statements relating to a topic (Watts & Stenner, 2005). Although the use of predetermined statements bounds subjectivity, each participant is free to sort the statements in the way that fits with their viewpoint at that moment in time.

**Figure 1.**
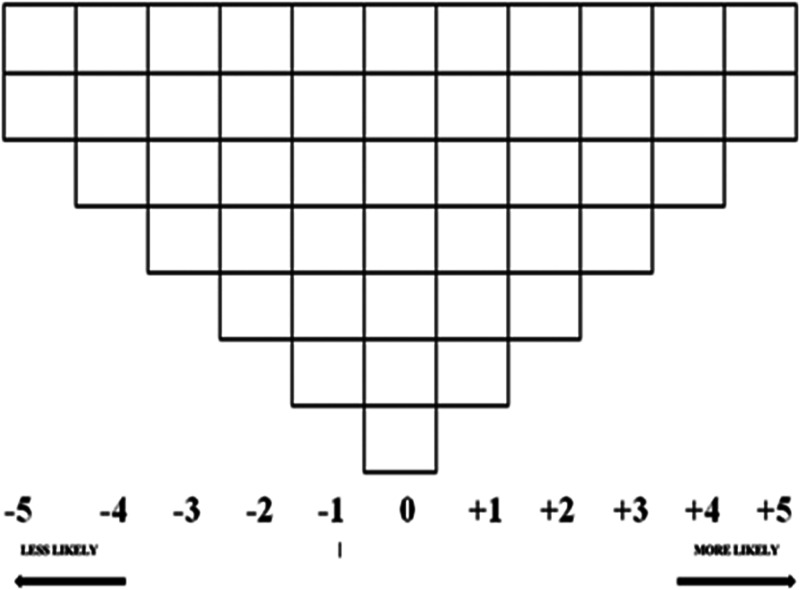
Sorting grid.

A Q-methodology study requires a statement concourse that adequately represents the focal topic. The statement concourse utilised here was derived from thematic analysis of qualitative data about disclosure in therapy provided by 120 adults with eating difficulties who took part in a mixed-methods study, as well as a review of the disclosure literature. This process resulted in a total of 192 statements about self-disclosure. Of these, 47 statements were utilised, the number being determined by the size and shape of the sorting grid (Figure 1). Statements were chosen to represent the different disclosure-related themes evident in the qualitative data and literature review. This included emotions related to disclosure (e.g. “feeling ashamed”), therapeutic relationship (e.g. “not trusting my therapist”), therapist self-disclosure (e.g. “not knowing much about my therapist”), therapist expertise (e.g. “thinking the therapist did not have enough knowledge”), consequences (e.g. “worried I might be admitted to hospital”), relationship to problems (e.g. “feeling my eating disorder was part of me”), and therapist reactions (e.g. “being judged by my therapist”). These statements had been used in a previous study with adults. Four young people aged between 16 and 19 from a child and adolescent mental health services participation group reviewed the statements to assess if they seemed clear and made sense.

### Participants

Participants were recruited from a specialist eating disorder service and a generic child and adolescent mental health service. Inclusion criteria were: (1) aged between 16 and 19; (2) have engaged in psychological therapy (current or in the past) for EDs and had at least two intervention sessions; (3) UK resident. It was not a requirement for participants to have a formal diagnosis of an eating disorder at the time of the study. A total of 37 people expressed interest in participating and all were eligible. Of these 28 (76%) completed the study. All were given a retail voucher in thanks for their participation.

### Materials and procedure

Participants received study materials by post. This included an information sheet, consent form, demographics questionnaire, instructions for completing the sorting task, sorting grid, 47 numbered statements on individual cards, a debrief sheet, and a prepaid envelope. The task instructions advised participants to consider their current experience of psychological therapy or, if they were no longer in therapy, their most recent experience.

Participants were first instructed to sort the statement cards into three piles related to their likely influence on disclosure: (1) more likely to influence; (2) less likely to influence; and, (3) neutral (neither more or less likely to influence their decision). They were asked to place them on the sorting grid (Figure 1) along a dimension ranging from −5 (less likely) to +5 (more likely) beginning first with the ‘more likely’ pile, then the ‘least likely’, and finally the ’neutral’ pile. The set shape of the grid, and the requirement of only one statement in each grid square, means that each statement is considered in *relative* likelihood to other statements. Participants were able to move the statements around until they felt satisfied with their arrangement. Participants were then asked to transfer the number on each statement card onto the corresponding square in the grid. Participants were also invited to note down any thoughts about the topic, their sorting, or the task generally on a separate sheet of paper and to return this with their completed grid and consent form.

### Data analysis and interpretation

Ken-Q Analysis Desktop Edition (KADE; Banasick, 2019) was used to analyse the Q-sorts. The factor extraction method was Horst Centroid and varimax rotation was applied. The analysis generates factors onto which participants load, with a rule of thumb being to select factors that have an eigenvalue >1. Participants who have a similar configuration of sorted statements will load onto the same factor. In determining a significant participant loading on a factor, Watts and Stenner (2005) provide a formula for calculating the minimum size of factor loading at *p* < .01 which is based on the number of statements in the sort. Utilising this formula, participant sorts were considered to load significantly on the factor when the loading was at least +/− .38. Any sorts loading on more than one factor at this level are considered to be confounding sorts and are not included in factor interpretation. Each factor extracted is represented by an exemplar factor array that demonstrates the overall viewpoint of the individuals whose sorts load significantly onto that factor. This provides the basis for factor interpretation.

The exemplar factor arrays are interpreted with the aim being to explicate the nuances of the viewpoint. Given the impossibility of interpreting more than 40 statements collectively, interpretation focussed initially on the two poles of the array (i.e. more/less likely to influence disclosure decisions) as suggested by Q-methodologists. Interpretation also involved a focus on distinguishing statements (i.e., those that most typify one factor but not others). Additionally, proximal statements were used to clarify the interpretation (Shemmings, 2006). Interpretation was also informed by participants’ additional comments and relevant literature. Each factor was given a label to represent the essence of the viewpoint. A correlation matrix between factors was examined to assess their distinctiveness. Demographic characteristics of participants loading on each factor were tabulated.

## Results

Most participants identified as female (*N* = 26; 93%). Age ranged from 16-19 years (M16.7, SD .95). Most were educated up to UK GCSE level (68%) or A-level/equivalent (25%). Two had no formal qualifications. Most were single (82%) and currently receiving treatment (82%; 54% prescribed medication). Anorexia Nervosa was the most common current diagnosis (*N* = 23; 88%). Two had a Bulimia Nervosa diagnosis, and one EDNOS. Two participants had not been diagnosed. Time since diagnosis was one to five years for most (*N* = 16; 57%), with the remaining ten diagnosed less than a year ago. CBT was the most common current intervention (50%), followed by counselling (46%) and family therapy (29%).

Three factors had an eigenvalue >1 and explained 42% of the variance. Three sorts did not load on any factor and seven were confounded. Therefore, 18 participant sorts contributed towards factor interpretation. There were generally small (r = .06, .17, .29) and non-significant correlations between the factors suggesting they were distinct. The analysis indicated that Factors 1 and 3 were bipolar. A bipolar factor is one in which two opposing viewpoints are expressed with each factor array being a mirror-image of the other. The interpretation here focuses on the array of positively loading sorts with an exact reverse interpretation reflecting the negatively loading sorts.

Table 1 presents the statements rated most and least influential to disclosure in the factor arrays. The distinguishing statements in each factor are also indicated along with the number of participant sorts loading on each factor. Following this, an interpretation of each factor is offered.

**Table 1.** Statements rated most and least influential in the factor arrays.

	Most influential to disclosure (Q-ratings 5 and 4)	Least influential to disclosure (Q ratings −4 and −5)
Factor 1 (*N* = 5; 19% variance): Knowing the therapist influences my decision more than not wanting to change^a^	5 Therapist telling me about similar experience they’ve had*	−4 No connection with my therapist
	5 Knowing information about my therapist*	−4 Feeling my problems were not bad enough*
	4 Therapist telling me they have had an eating disorder*	−4 Not wanting to change*
	4 Therapist telling me details of their life experiences*	−5 Not trusting my therapist
	4 Feeling worried my treatment would be discontinued*	−5 Feeling eating disorder a part of me I didn’t want to lose*
Factor 2 (*N* = 5; 14% variance): Maintaining my ideal influences my decision more than knowing the therapist or them being qualified to help	5 Wanting to be perfect*	−4 Therapist telling me about similar experience they’ve had*
	5 Not wanting to change	−4 Therapist telling me they have had an eating disorder*
	4 Feeling eating disorder a part of me I didn’t want to lose	−4 Fearing I might damage my therapist
	4 Feeling my problems were not bad enough*	−5 Thinking the therapist did not have enough knowledge*
	4 Looking like an attention seeker*	−5 Thinking the therapist did not have appropriate training*
Factor 3 (*N* = 4; 9% variance): Keeping information to myself influences my decision more than negative therapist reactions or consequences^a^	5 Wanting to keep information a secret*	−4 Knowing information about my therapist
	5 Not wanting to change	−4 Feeling worried I might be admitted to hospital*
	4 Not wanting my therapist to know everything*	−4 Wanting to be perfect*
	4 Not liking my therapist*	−5 Feeling worried my therapist may become angry*
	4 Feeling the therapist did not understand me*	−5 Fearing I might damage my therapist*

*Note.* *Distinguishing statement for that factor; ^a^bipolar factor – negatively loading sorts are the mirror image of factor and have reverse interpretation (Factor 1 *N* = 3; Factor 3 *N* = 1).

Table 1 indicates the existence of three distinct factors. Whilst statements reflecting the influence of wishing to maintain eating difficulties feature in Factors 1 and 2, they are rated as least influential in Factor 1 (positive array) but most influential in Factor 1 negative array and Factor 2. However, examining specific items and distinguishing statements reveals nuances between these latter two factors. For example, the maintenance of difficulties reflects a wish to be perfect in Factor 2 that is not evident in Factor 1 (negative array). Factor 3 contrasts notably with the others in terms of distinguishing statements and its overall essence.

### Factor 1: Knowing the therapist influences my decision more than not wanting to change

The decision to disclose is most strongly influenced by finding out information about the therapist, including them having an eating disorder specifically, and similar experiences more generally. All of these were distinguishing statements. The distinguishing statement regarding treatment discontinuation helps to nuance the interpretation as does the comment of a participant who noted “*I was worried I would not be taken seriously*”. Taken together, these data potentially reflect a concern to know if the therapist has sufficient experiential similarity such that participants’ difficulties will resonate with them. The idea of wanting to engage in treatment is substantiated further by the items rated as being least influential, three of which were distinguishing statements relating to resistance to change.

Notably, aspects relating to connection with and trust in the therapist were also rated as least influential. The stronger influence of gaining personal knowledge of the therapist relative to the having a connection and trust might seem counterintuitive, unless interpreted in the context of the preceding interpretation which suggests that the way to trust is first through knowing sufficient information about the therapist. In a Q-sort, the items are placed relative to one another and are not indicative of some aspects of the topic being absolutely uninfluential. The interpretation above is supported by literature suggesting the need to establish evidence of trust and secure care are key to recovery in anorexia nervosa (Karlsson et al., 2021). Obtaining disclosures is one way to gather such evidence. These views around therapist disclosure might also be interpreted through the lens of the motivation to retain control which is particularly significant for people with this diagnosis (Bryant et al., 2022). For some, connection and trust might seem secondary to the need for control in a disclosure context. The importance of control is also indicated by proximal statements; wanting to be perfect, concerns about vulnerability, and being left with difficult feelings were rated as more influential (+3). In summary, this factor suggests disclosure decisions are influenced more by knowledge and control of feelings and less by not wanting to change.

### Factor 2: Maintaining my ideal influences my decision more than knowing the therapist or them being qualified to help

The strongest influences on disclosure decisions in this factor centred on maintaining an ideal self. A nuance was not only an emphasis on being perfect but also on presentation of self to others both of which were distinguishing aspects of this factor. The therapist has the least influence on disclosure decisions, pertaining not only to who they are but how good they are, and whether they might get harmed in the relationship. This factor has surface coherence; if a person does not want to change, it is likely they will be relatively less influenced by perceived therapist competence. As one participant noted *“Generally my answers were based on not wanting to change”.* Supporting the need to maintain self-integrity, proximal statements rated as influential included fears of being admitted to hospital, not feeling ready (10: +3), and feeling embarrassed (all rated +3). An interpretation that self-protection may be particularly important for these participants is supported by comments written by participants e.g. “*Behaviours I really struggle with I would not mention due to embarrassment and feeling awkward”,* and *“I find it difficult to open up because I am accepting that there is in fact an issue. Once I say it out loud, I can talk more but only because I have crossed that hurdle. The guilt builds up inside and the longer it is repressed the harder it is to open up to anyone”.* Further reinforcing the centrality of self and relative lack of influence of the therapeutic relationship proximal statements of least influence included not having a rapport, no connection, and not liking the therapist (all rated −3).

A recent phenomenological meta-synthesis of qualitative research conducted over the past two decades evidences the ways in which anorexia nervosa is intertwined with identity and the functions it serves in terms of self-protection and meaning (Bryant et al., 2022). Stronger concerns about self-protection and maintenance of ED identity are likely to overshadow the influence of having a relationship with someone whose agenda is focussed on facilitating change. In addition to aspects relating to maintaining identity and not wanting to change, statements related to fear of admission to hospital or being sectioned also connect with the literature. Hospitalisation and enforced treatment are strong threats to self-control, the battle over which is noted as a key aspect of anorexia phenomenology (Bryant et al., 2022).

### Factor 3: Keeping information to myself influences my decision more than negative therapist reactions or consequences

Similarly to Factor 2, this factor conveys a sense of self-integrity as being most influential albeit expressed differently. Whilst both factors have not wanting to change as highly influential to the decision to disclose, there is divergence in terms of the nature of self-protection. For those loading positively on this factor, strong influences on disclosure decisions relate to wanting to keep information from the therapist. An interesting parallel here is that the statement related to knowing information about the therapist was one rated as least influential. Other statements rated as having a strong influence further contextualise the desire for secrecy and withholding. Statements about not liking the therapist and feeling the therapist does not understand were influential distinguishing statements. Not trusting the therapist was also influential (rated +3). Participant comments support the importance of understanding, for example *“Before I came to CAMHS I felt misunderstood’.*

The least influential statements in this factor related to potential strong emotions that might be evoked in the therapist or other consequences that might promote relationship rupture or termination. This is an interesting contrast to Factor 2 in which the relatively less influential relationship factors related to experience and competence, and for Factor 1 trust and connection. What all three have in common though is a seeming demotion of the relative importance of the relationship and the centring of self-protection in relation to disclosure decisions. Withholding information from the therapist may be motivated by the desire to avoid shame. Swan and Andrews (2003) found that almost a half of women who had experienced eating disorders had withheld information in therapy and that both characterological shame and that related to eating was significantly associated with non-disclosure. This context helps to understand the significance of not liking the therapist and not feeling understood as other strongly influential factors in this factor.

### Per factor demographic characteristics

Generally, the characteristics of participants loading onto each factor are similar (Table 2). As such, these data do not suggest that aspects of self or ED profile differentiate participants who loaded onto the different factors.

**Table 2.** Characteristics of participants loading onto each factor.

Factor	Age	Gender and ethnicity	Education level	Relationship status - single	Currently in treatment	Diagnoses received	Time since diagnosis
Factor 1 (*N* = 5): Knowing the therapist influences my decision more than not wanting to change	16.0	Female White British	4 GCSE 1 A-Level	4 of 5	All	4 AN, 1 EDNOS	2 <1 year 3 1–5 years
Factor 1 Reverse viewpoint: (*N* = 3)	17.3	Female White British	1 GCSE 2 A-Level	2 of 3	2 of 3	AN	1 <1 year 2 1–5 years
Factor 2 (*N* = 5): Maintaining my ideal influences my decision more than knowing the therapist or them being qualified to help	16.4	Female White British	4 GCSE 1 A-Level	3 of 5	4 of 5	4 AN 1 BN	1 <1 year 3 1–5 years
Factor 3 (*N* = 4): Keeping information to myself influences my decision more than negative therapist reactions or consequences	17	Female White British	3 GCSE 1 A-Level	3 of 4	3 of 4	AN	1 <1 year 3 1–5 years
Factor 3 Reverse viewpoint (*N* = 1)	17	Female White British	No formal qualification	Yes	Yes	EDNOS	<1 year

## Discussion

Non-disclosure of disordered eating by young people is a complex and demanding situation for clinicians to manage and may reflect defensive denial or conscious withholding. Defensive denial might arise where the eating disorder has become a part of the young person’s identity; disclosure may threaten the very integrity of self. Conscious withholding may be motivated by shame and fear of labelling, stigmatisation, and loss of control (Howard et al., 2023). Such powerful motivations for non-disclosure are likely to result in significant tension building within the therapeutic relationship. Clinicians need skills and support to tolerate lack of disclosure, perhaps for a protracted period of time, while simultaneously managing risk for a young person who may be deteriorating medically and developing a trusting therapeutic relationship.

Aspects of what might broadly be termed the therapeutic relationship were defining statements in all three factors. However, the findings indicate that aspects of the therapeutic relationship, including sense of connection and trust, were perceived to be relatively less central for some participants than others. For some, the ability to gain personal knowledge of the therapist was of more importance whilst, for others, self-protection, readiness, and willingness to change had relatively greater emphasis than aspects of the therapeutic relationship. The value of Q-methodology lies in understanding what is relatively more or less important in a holistic sense for different people at different times; this has practical significance. For example, for some the influence of the therapeutic relationship on disclosure decisions will be secondary to the prominence of knowing information about their therapist.

The observed differential viewpoints regarding the influence of therapist self-disclosure connect to a substantial corpus of work albeit the majority being based on adults. Whilst the literature might previously have discouraged this practice, more contemporary literature acknowledges that judicious disclosure regarding the therapist’s professional and/or personal experience might enhance trust within the therapeutic relationship (de Vos et al., 2016). Going further than this, Gaines (2003) discusses the necessity of therapist self-disclosure in the process of reducing a young person’s anxiety and facilitating communication, both of which are required if a therapeutic relationship is to be established. Similarly, Tantillo (2010) argued that therapist self-disclosure is particularly important when working with adolescents because they are in a developmental period where self-individuation in relationships with others is particularly key. Through self-disclosure, therapists can model that it is acceptable to share difficult feelings and behaviours, and can stimulate a sense of empathy that supports adolescents to allow themselves to be vulnerable and open with others. In contrast, the factors in which therapist self-disclosure was de-emphasised may connect with the literature that indicates variability in the perceived usefulness and appropriateness of therapist self-disclosure (Simonds & Spokes, 2017). However, it is important to bear in mind that ranking in a Q-sort task indicates relative rather than absolute importance; it is not that therapist self-disclosure is not influential, it is relatively less so.

Individuals who held the viewpoint that therapist self-disclosure was more influential in their decision to disclose and who appeared more accepting of the need for change may benefit from a more contemporary relational psychoanalytic approach to treatment, as proposed by Gaines (2003). He suggests that adolescents are more flexible than adults in terms of their ability to change their behaviour and their experience of self through the development of new relationships. He argued that self-disclosure can help adolescents to see that the therapist is not like the “old objects”, instead creating a new relational experience for them. He also proposed that characteristics that we relate to in a good relationship such as empathy, genuineness, care, and respect can be improved by the sensitive use of therapist self-disclosure. This may make it easier for this group to let go of their eating difficulties as they develop a greater level of self-assurance through the therapeutic relationship and an acceptance of their authentic self.

For others, holding on to their idealised self or wanting to hold information away from the therapist seemed to be important in making a decision to self-disclose. Client self-disclosure in therapy could help to re-establish fragmented parts of identity and reinforce those parts that have become lost (Farber, 2006). This could support the development of a more cohesive sense of self and allow relinquishment of the need for an ideal self that masks a ‘true self’ (Williams et al., 2016). A treatment approach that supports the externalisation of the eating problems in an attempt to support the individual to disentangle from their eating-focussed identity to give way for other identities to develop and become more prominent may be helpful for this group. A Narrative Therapy model (Epston & White, 1992) seeks to do this, encouraging the individual to see their self as distinct from the eating disorder entity (Morgan, 2000). The focus of this approach would be to deconstruct the most salient narrative that is keeping the individual in the grips of the eating difficulties and replace it with a richer narrative based on the person’s values and goals that have become lost under the power of the disorder (Morgan, 2000). Maisel et al., (2004) stated that supporting individuals to develop alternative narratives about themselves supports them to change their beliefs surrounding their self-worth and helps them to reconnect with values not associated with their eating.

Given that CBT and family-based interventions currently hold the biggest evidence base for the treatment of eating difficulties in adolescents in the UK, it would be unrealistic to suggest that services should radically change the interventions that are offered or provide more longer-term psychodynamic interventions. However, ideas from the narrative approach and more contemporary psychoanalytic approaches that consider issues around identity and the more relational aspects of therapy could be considered and integrated into shorter-term interventions.

It is important to consider ways in which motivations for non-disclosure might be assessed prior to any therapeutic intervention. They are likely to be linked to motivations and readiness to change, the evaluation of which is central to any proposed intervention (Zimmerman et al., 2000). The Eating Disorder Readiness Ruler may be one way of assessing this (Treasure et al., 2016). This simple self-report instrument assesses readiness to change eating disorder behaviours and has been shown to have potential clinical utility (St-Hilaire et al., 2017). Motivational interviewing could also be used as a tool to increase desire to change and potentially enhance disclosure (Miller & Rollnick, 2002).

Whilst the current study has provided a new insight into the processes involved in self-disclosure decisions, future research could enhance understanding. It would be of value to conduct longitudinal studies to ascertain how influences on disclosure decisions change during different phases of recovery. Moreover, in-depth qualitative data could be collected to gain a deeper understanding of participant sorting decisions. Research has shown that in the last decade there has been a rise in new cases of anorexia in children aged 8–12 (Petkova et al., 2019). Extending this research to a younger population would therefore be of benefit to facilitate early intervention. Furthermore, the study sample was predominantly White British females. Research should explore factors that influence self-disclosure in a more diverse sample. There was also limited variability in the profile of participants’ eating difficulties which should be expanded in future research.
